# Insights into Structural Modifications of Valproic Acid and Their Pharmacological Profile

**DOI:** 10.3390/molecules27010104

**Published:** 2021-12-24

**Authors:** Manish Kumar Mishra, Samiksha Kukal, Priyanka Rani Paul, Shivangi Bora, Anju Singh, Shrikant Kukreti, Luciano Saso, Karthikeyan Muthusamy, Yasha Hasija, Ritushree Kukreti

**Affiliations:** 1 Genomics and Molecular Medicine Unit, Institute of Genomics and Integrative Biology (IGIB), Council of Scientific and Industrial Research (CSIR), Mall Road, Delhi 110007, India; manishkrmishra18@gmail.com (M.K.M.); samiksha@igib.in (S.K.); priyankapaul008@gmail.com (P.R.P.); shivee30@gmail.com (S.B.); 2 Department of Biotechnology, Delhi Technological University, Shahbad Daulatpur, Main Bawana Road, Delhi 110042, India; yashahasija06@gmail.com; 3 Academy of Scientific and Innovative Research (AcSIR), Ghaziabad 201002, India; 4 Nucleic Acids Research Lab, Department of Chemistry, University of Delhi (North Campus), Delhi 110007, India; anju11278@gmail.com (A.S.); skukreti@chemistry.du.ac.in (S.K.); 5 Department of Chemistry, Ramjas College, University of Delhi (North Campus), Delhi 110007, India; 6 Department of Physiology and Pharmacology “Vittorio Erspamer”, Sapienza University of Rome, P. le Aldo Moro 5, 00185 Rome, Italy; luciano.saso@uniroma1.it; 7 Department of Bioinformatics, Alagappa University, Karaikudi 630004, Tamil Nadu, India; mkbioinformatics@gmail.com

**Keywords:** valproic acid, anticonvulsant, analogues, derivatives, teratogenicity, hepatotoxicity, development

## Abstract

Valproic acid (VPA) is a well-established anticonvulsant drug discovered serendipitously and marketed for the treatment of epilepsy, migraine, bipolar disorder and neuropathic pain. Apart from this, VPA has potential therapeutic applications in other central nervous system (CNS) disorders and in various cancer types. Since the discovery of its anticonvulsant activity, substantial efforts have been made to develop structural analogues and derivatives in an attempt to increase potency and decrease adverse side effects, the most significant being teratogenicity and hepatotoxicity. Most of these compounds have shown reduced toxicity with improved potency. The simple structure of VPA offers a great advantage to its modification. This review briefly discusses the pharmacology and molecular targets of VPA. The article then elaborates on the structural modifications in VPA including amide-derivatives, acid and cyclic analogues, urea derivatives and pro-drugs, and compares their pharmacological profile with that of the parent molecule. The current challenges for the clinical use of these derivatives are also discussed. The review is expected to provide necessary knowledgebase for the further development of VPA-derived compounds.

## 1. Introduction

Valproic acid (VPA, 2-propyl-pentanoic acid), is a fatty acid derivative of valeric acid naturally found in *Valeriana officinalis* [1,2]. Synthesized in 1881 by Beverly S Burton [3], VPA continued to be used as an organic solvent till 1963 before its role as an anticonvulsant was discovered serendipitously by Meunier et al. [4]. Following its first marketing as an antiepileptic drug (AED) in France, VPA represents one of the most efficient and widely prescribed AED world-wide [1]. With its recognized role as an AED, VPA has drastically evolved as a molecule targeting a spectrum of pathologies such as bipolar disorder, migraines and neuropathic pain [5,6,7]. VPA has shown promise as a neuroprotective agent in Alzheimer’s disease and multiple sclerosis [8,9,10]. Additionally, overlapping pathways and interactions have led to its recognition as a compound of interest in the clinical trials for a wide variety of cancers [11,12,13,14]. However, the potential adverse effects associated with VPA use are hepatotoxicity and teratogenicity, which interfere with its therapeutic use [15]. Substantial experimentation and research have gone into elimination of these adverse effects and to improve its efficacy by introducing structural changes in the VPA skeletal structure. These changes ranged from branching and alpha fluorination to production of chiral compounds, from its amide derivatives to cyclic derivatives and conjugation products. This article briefly discusses on the structural elements of valproic acid, its pharmacokinetics and pharmacodynamic profile. The focus of the review is to provide a comprehensive account of all the derivatives and analogues of VPA and ascertain their success as possible replacement of the parent compound as better, safe and tolerable drugs. Any compound second generation to VPA shall address the current challenges of potency and teratogenicity along with hepatotoxicity of the metabolites. This knowledge of the existing modifications will serve a platform to design better successors of VPA.

## 2. Structure and Pharmacology of Valproic Acid

### 2.1. VPA Structural Elements

The simple chemical structure of VPA makes it unique in the arsenal of drugs that are used for same treatments as VPA. It is an eight-carbon molecule with the backbone of pentanoic acid and a propyl group attached to the second carbon. It is a weak organic acid with pKa value of 4.8, that causes 99.8% ionization of the molecule at physiological pH [16]. Therefore, despite of good lipophilicity (logP = 2.75) it requires carrier protein for its cellular uptake [16]. Keane et al., reported lack of anticonvulsant activity of non-branched analogues of VPA in vivo [17]. On the contrary, branched analogues were found to significantly increase the brain concentration of gamma-aminobutyric acid (GABA), showing anticonvulsant activity against induced seizures in animal models [17,18,19]. These studies indicated the essentiality of the branched structure of VPA for anticonvulsant activity. It has also been proposed that C=O group is the main functional group controlling the anticonvulsant behavior by providing the initial electrostatic interaction between VPA and the target protein [20]. Other than the molecule’s therapeutic effect, structure-teratogenicity relationship studies performed in mouse strains implicated that the tetrahedral structure, the hydrogen attached to C-2 and aliphatic side chain branching [21] may be responsible for the undesirable teratogenic effect associated with its use [22]. Lloyd et al., also suggested the role of carboxylic acid moiety in causing neural tube defect. It has been reported that the carboxylic group of VPA that exists as an anion at physiological pH interacts with organic anion transporters (OATs) present on the foetal facing/basolateral plasma membrane of the placenta [23]. This interaction of VPA with OATs leads to VPA accumulation in foetus causing embryotoxicity [23]. Next substantial side-effect, hepatotoxicity is primarily caused by metabolites of VPA (4-ene-VPA and 2,4-diene-VPA) [24,25,26,27].

### 2.2. VPA Pharmacokinetics

Valproic acid is available for treatment in various forms such as oral solutions, tablets, enteric-coated capsule and intravenous solution, with bioavailability up to 100% and absorption half-life ranging from 30 min to 4 h depending upon the type of formulation used [28,29]. Among the present preparations, the oral formulations are widely used with enteric coated capsule showing highest bio-availability compared to other oral formulations [28]. Food intake influences the absorption of VPA, with absorption being slowest when taken after three hours of a meal as compared to when taken with meals or during fasting conditions [30].

Established metabolic routes of VPA are glucuronidation and beta oxidation, which account for 50% and 40% of VPA metabolism, respectively [31,32]. Studies have also explored the involvement of cytochrome P450 (CYP450)-mediated oxidation, although its contribution remains low (~10% VPA metabolism) [33,34]. A major metabolite found in urine samples is a conjugate product of VPA and glucuronic acid (15% to 40% of total VPA administered in humans) [35,36]. The metabolites produced via oxidation pathways are known to cause hepatotoxicity. Direct esterification of VPA or esterification of one of the major metabolites, 4-ene-VPA, forms respective acetyl-CoA esters [37,38]. These esters are highly reactive and they form thiol conjugates with mitochondrial glutathione, which result in the depletion of mitochondrial glutathione. The resulting reduction in the glutathione pool causes interference with mitochondrial function, which leads to hepatotoxicity [37,39,40].

The plasma level of a free fraction of VPA therapeutic concentration is 6%; the rest is bound to protein mainly albumin. Due to its high affinity for the protein, VPA shows a low clearance rate of 6–8 mL/h/kg [41,42,43].

### 2.3. VPA Pharmacodynamics

Valproic acid is primarily prescribed for the treatment of epilepsy, bipolar disorder and migraines. The anticonvulsant property of VPA is due to its influence on brain concentrations of GABA. It indirectly inhibits GABA degradation by hindering the activity of enzymes, such as GABA transaminase (GABA-T) and succinate semialdehyde dehydrogenase (SSADH) that are involved in GABA decay [44]. Furthermore, VPA acts on GABA-receptor to potentiate postsynaptic inhibitory responses [29].

**Figure 1:**
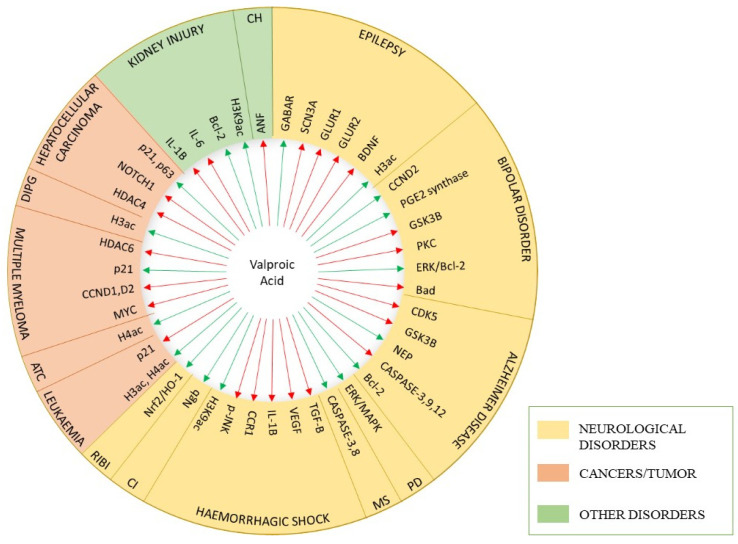
Molecular targets of valproic acid. Different targets regulated by valproic acid are represented by arrows for neurological disorders (epilepsy, bipolar disorder, Alzheimer’s disease, Parkinson’s disease (PD), encephalomyelitis), haemorrhagic shock, ischemia, injury (radiation-induced brain injury (RIBI), kidney injury), cardiac hypertrophy (CH) and cancers (leukaemia, anaplastic thyroid carcinoma (ATC), multiple myeloma, diffuse intrinsic pontine glioma (DIPG), hepatocellular carcinoma). The green arrows indicate upregulation of expression and red arrows indicate inhibition/downregulation of target gene.

Other than affecting inhibitory synapses, VPA has been demonstrated to reduce the neuron surface expression and synaptic localization of glutamate-receptor subunits, GluR1/2, thus preventing excitatory effect [29]. It also obstructs various voltage gated ion channels such as Na^+^ channels, voltage-gated K^+^ channels, and voltage-gated Ca^2+^ channels [45]. Such an obstruction relaxes the high frequency neuronal firing [46,47]. The downregulation of sodium voltage-gated channel alpha subunit 3 (SCN3a) expression by reducing the gene promoter methylation in mouse neural crest-derived cells is also suggested as an epigenetic pathway in the anticonvulsant role of VPA [48]. More recent finding of VPA pharmacodynamics is its role as histone deacetylase (HDAC) inhibitor [12,13]. leading to increase in gene expression of apoptotic proteins and hence its activity in cancer treatment [14]. Apart from these mechanisms, evidence demonstrates that VPA affects other signaling systems to exert its antimanic, neuroprotective, anti-inflammatory and antioxidant effect, which may lead to extension of its use in other non-epileptic neurological disorders; however, discussing these diverse roles is beyond the scope of this article. Figure 1 summarizes major molecular targets of VPA for various therapeutic areas.

## 3. Structural Modification of Valproic Acid: Derivatives and Analogues

**Figure 2:**
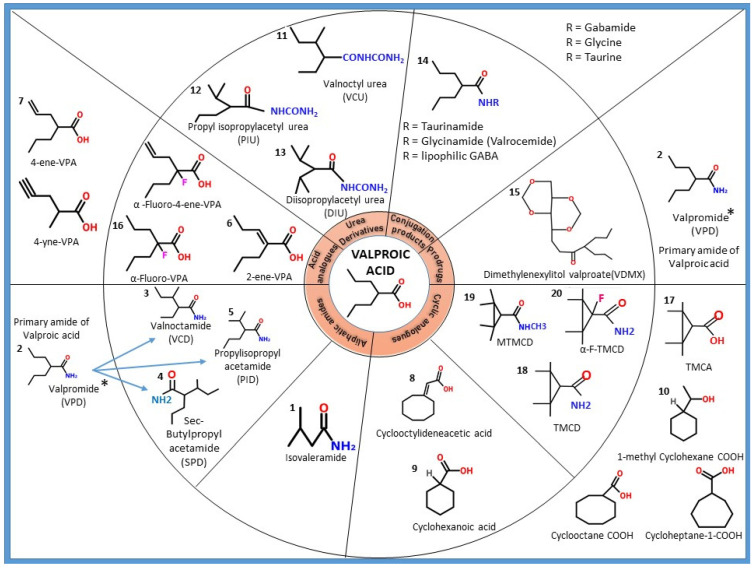
Schematic representation of valproic acid derivatives and analogues. These compounds are characterised into aliphatic amides, acid analogues, conjugation products, prodrugs and cyclic analogues. The aliphatic amides are further characterised into chiral and achiral compounds, and acid analogues are further characterised into unsaturated and alpha fluorinated compounds. The compounds outside the circle are those with some limitations ceasing their further study. The compounds included in the circle are those that are under active investigation. * Valpromide is mentioned twice since along with being a primary amide of valpromide; it also behaves as a prodrug.

In attempts to minimize the adverse effects associated with VPA use and to improve its potency, various analogues and derivatives have been synthesized and studied in comparison to the parent molecule. As shown in Figure 2, primary modifications include altering the carboxylic acid group (replacing it with the amide); altering the main chain (replacing with cyclic entities) and/or changing the branching pattern by introducing unsaturated bonds. This section elaborates the literature reported under each VPA derivative describing the structural changes to the parent molecule, comparison of pharmacological properties in terms of potency, activity and toxicity profile and the clinical applicability of the compound. A brief summary of the above is shown in Table 1.

### 3.1. Amide Derivatives of VPA

Amide derivatives of VPA have proven themselves to reduce the hepatotoxicity and teratogenicity of the parent compound. They have also depicted improved potency except valpromide, which systemically bio-transforms into VPA in human subjects [66].

#### 3.1.1. Isovaleramide

Isovaleramide, a branched chain amide derivative of isovaleric acid (naturally occurring VPA analogue) with the molecular weight of 101.149 g/mol was developed by NPS Pharmaceuticals Inc., Salt Lake City, UT, USA under the alias of NPS 1776 [49]. Isovaleramide has a palatable taste and odour, unlike its parent compound isovaleric acid, which has an undesirable taste and odour [49]. Though, no mechanism of action for this compound has been reported, it is targeted for treatment of epilepsy and migraine attacks, and has shown broad spectrum of anticonvulsant activity in maximal electroshock stimulation (MES)-, pentylenetetrazole (PTZ)-induced seizures and kindling models along with chemo-convulsant and absence epilepsy models [49].

Islovaleramide exhibits linear pharmacokinetics in the dose range of 100–1600 mg [49]. The drug was found to be safe and well tolerated up to maximum dose of 2400 mg per day administered to phase 1 human volunteers with none of the adverse effects associated with VPA use [49]. Isovaleramide does not show any inhibitory effect on CYP450 enzymes, which may prevent any significant drug–drug interactions [49]. One limitation of the compound is its lower half-life of just 2.5 h, which leads to its rapid elimination. The reduced half-life of this potential drug can be targeted by making conjugation products or sustained release formulations [49]. Currently, the drug is undergoing phase II clinical trials for treatment of acute migraine headaches [67].

#### 3.1.2. Valpromide (VPD)

Valpromide (VPD, dipropylacetamide, 2-propylvaleramide) is a 143.23 g/mol primary fatty acid amide of VPA, differing by the presence of amide group replacing the carboxylic acid functional group. This substitution makes it a more potent AED (2–5 times) as observed in in vivo models [68].

As opposed to the HDAC inhibitory effect of VPA, studies prove that VPD does not interfere with HDAC activity and thus has a highly diminished teratogenic effect in animals [12,69]. A study by France-Hélène Paradis and Barbara F. Hales [70] demonstrated that VPA causes downregulation of both SRY-Box Transcription Factor 9 (SOX9) and Runt-related transcription factor 2 (RUNX2)-signalling pathways involved in chondrogenesis and osteogenesis, leading to an overall teratogenic limb malformation in rat embryos. The downregulation of these pathways was likely the consequence of histone-4 hyperacetylation by VPA. VPD essentially failed at affecting histone 4 hyperacetylation or SOX9 expression, with only minimally altering the expression of RUNX2, thus lacking teratogenic potential. However, VPD proved to be a stronger sedative and neurotoxin than VPA in a mice model of seizures [68].

Reports show that valpromide gets partially converted to valproic acid in dogs and rats [71]. Consistent with this, Bailer et al. found that following the oral administration of VPD in human subjects, blood plasma detection of VPA was observed instead of VPD. This suggested that VPD acts as a prodrug to VPA in humans, and bio-transforms into VPA in humans, due to which its superiority over VPA in vivo does not have any clinical significance [72].

To build upon the reduced teratogenicity, improved potency and to resist metabolic hydrolysis of the amide to its parent carboxylic acid compound, a further three compounds were derived from VPD (valnoctamide, sec-butyl-propyl carboxamide and propylisopropyl acetamide) [56].

##### Valnoctamide (VCD, 2-Ethyl-3-methylpentanamide)

Valnoctamide is a 143.23 g/mol constitutional isomer of VPD with valnoctic acid (VCA) as its corresponding acid. Structurally, the parent chain has two branches instead of one making two chiral centres. The first branching is from an ethyl group at C-2 position and the second branching is from a methyl group at C-3 position. Similar to VPD, VCD has also shown higher potency than VPA in rodent models, which might be attributed to its better brain distribution [50]. In a pilocarpine-treated rat model of focal epilepsy, VCD proved as effective as its parent compound VPD and its analogue VPA in showing protective and antiepileptogenic effects [73], although it failed to show any activity in status epilepticus (SE) model [51]. Additionally, both VCD and VPD also demonstrated improved attenuation of extracellular glutamate levels as compared to VPA [73].

Apart from anticonvulsant action, the enantiomers of VCD have been demonstrated to show efficacious antiallodynic activity in rats with neuropathic pain induced by spinal nerve ligation (SNL) [74]. Of the two diastereoisomers, (2*S*,3*S*)-VCD demonstrated lesser embryotoxicity and higher potency than (2*R*,3*S*)-VCD, indicating that (2*S*,3*S*)-VCD as the potential molecule for neuropathic pain treatment [74].

VCD was found to have longer half-life and mean residence time than VPD [50]. Besides, unlike VPD, VCD does not bio-transform into its corresponding acid or VPA under physiological condition and thus has a greatly reduced teratogenic potential. Okada et al. reported that inhibition of Polycomb group (Pc-G) gene expression by VPA led to axial skeletal abnormalities in embryos of pregnant NMRI mice, whereas VPD and VCD did not show any significant effect on these genes, thus proving them to be much less teratogenic than VPA [69]. The same group also revealed that VPA affects expression changes in genes involved in the cell cycle and apoptosis pathways of neural tube cells, causing neural tube defects in embryos of NMRI mice. However, VCD and VPD affected only few of these genes with majority of genes unaffected [75]. VCD has undergone phase III clinical trials and is primarily prescribed for mania, bipolar, and schizoaffective disorders [66,74]. Nirvanil, which is a racemic mixture of both enantiomeric pairs of VCD, is a marketed anxiolytic drug in some European countries, namely France, Holland, Italy, and Switzerland [76,77].

##### *sec*-Butyl-propylacetamide (SPD, 3-Methyl-2-propylpentanamide)

Another amide of VPA developed from VCD is its one-carbon homologue namely *sec*-Butyl-propylacetamide (SPD) and has been under investigation as a replacement of VPA. SPD differs from VCD by having a propyl rather than ethyl side chain at C-2 position. SPD demonstrated broad-spectrum antiseizure profile comparable to VPA [51]. Being currently in phase II clinical trials, SPD in animal studies has been effective against both focal and secondarily generalized seizures [51]. A dose-dependent decline was observed in secondarily generalized seizures when SPD was tested on kindled rodent models with median effective dose (ED_50_) values lesser than VCD and hence better efficacy [51]. It also caused a dip in seizure score from 4.6 to 1.3 in hippocampal kindled rats at a dose of 32 mg/kg and also with corneal kindled mice from 5 to 1 at 60 mg/kg [51]. In case of refractory 6Hz limbic seizures, an ED_50_ = 27 mg/kg for SPD was observed which is less than that of VCD (ED_50_ = 37 mg/kg) [51]. Moreover, unlike VCD, SPD also showed its activity in pilocarpine SE model with an ED_50_ value of 84 mg/kg with improved neuroprotective role as compared to VPA. SPD has also shown promising results in managing benzodiazepine-resistant seizures [51]. These findings implicate better efficacy of SPD over VCD.

Kaufmann et al. studied the antiallodynic activity of range of VPA related compounds in SNL model and found SPD to be as effective as Gabapentin in countering neuropathic pain with ED_50_ values of 49 mg/kg along with mild sedation observed at dose of 80 mg/kg [78].

The lack of teratogenicity was shown in studies where racemic SPD failed to induce neural tube defects (NTD) in SWV mice. Thus, along with better anticonvulsant property, it is nonembryotoxic and non-teratogenic [79].

##### Propylisopropyl Acetamide (PID, Diisopropyl Acetamide)

PID is a 143.23 g/mol constitutional isomer of VPD with a single stereocenter. As the name indicates, it has an isopropyl side chain at C-2 parent carbon instead of a n-propyl group as it is in VPA. It has anticonvulsant ability comparable to VPD and about 3–30 times more potency than VPA [80,81]. PID and VCD were tested for their anticonvulsant activity against generalized convulsive seizures in developing rats induced with PTZ. PID showed better control of generalized tonic–clonic seizures (GTCS) in 25 days old rats as compared to much younger ones, whereas VCD proved to be an overall better anticonvulsant, with no significant differences observed between the two stereoisomers [82]. Stereoselectivity of PID enantiomers was seen in animal models, where it was observed that the R enantiomer is a more potent anticonvulsant than S-PID with ED_50_ of R-PID being 19–32% lower than the latter [83]. It was also found to be more potent in 6Hz seizure model compared to S-PID [81].

The results of teratogenic effect of VPA and PID in pregnant NMRI mice exhibited that in contrast to VPA causing 37% and 73% (water and Cremophor EL suspension respectively) exencephaly in foetuses; PID and its enantiomers did not show any significant teratogenic effect [84].

The development of PID as second generation VPA was stopped due to financial limitations of the holding company Jazz Pharmaceuticals [85].

### 3.2. Acid Analogues of VPA

#### 3.2.1. 2-ene-VPA (2-Propyl-2-pentenoic Acid)

2-ene-VPA is a 142.2 g/mol, unsaturated metabolite of VPA with a double bond between C-2 and C-3 has undergone phase I clinical trial in humans to establish its safety and tolerability [53]. Its potency was observed to be 60–90% of the parent compound in electro and chemiconvulsive threshold tests in vivo [86,87]. Loscher et al. did the comparative analysis of 2-ene-VPA against the parent compound in mice models with MES and PTZ seizure, rats with chronically reoccurring spontaneous petit mal seizures and gerbils with GTCS [88]. The results of the study clearly reflected that 2-ene-VPA has comparable ED_50_ values to that of VPA and no toxicity at effective dose in gerbils and rats. The study also reported lack of embryotoxicity even at doses as high as 600 mg/kg [88]. However, 2-ene-VPA showed greater sedation than VPA at a dose of 200–300 mg/kg in MES- and PTZ-induced seizures [89].

The pharmacokinetic shortcoming of this analogue was its partial biotransformation into VPA, due to which its development as an AED was ceased [90].

#### 3.2.2. 4-ene-VPA (2-n-Propyl-4-pentenoic Acid)

4-ene-VPA is a 142.2 g/mol acidic analogue and metabolite formed by terminal desaturation of valproic acid, by the action of CYP2C9 [44]. It is shown to have anticonvulsant potency similar to VPA [86,87].

In a study comparing teratogenic potencies of R and S enantiomeric forms of 4-ene-VPA in NMRI mice, S-(-)-4-ene-VPA was revealed to show four times higher exencephaly compared to the R enantiomer and also had higher teratogenic potential than VPA itself [54]. 4-ene-VPA also demonstrated hepatotoxicity in rats, and proved to be a good prediction compound in urine to ascertain hepatotoxicity of VPA [27]. Thus, higher hepatoxicity and teratogenicity of the metabolite than VPA despite having similar anticonvulsant activity might reason the absence of its clinical trial record.

### 3.3. Fluorinated Derivatives

Alpha-fluorovalproic acid is a 162.2 g/mol fluorinated derivative of valproic acid. This derivatisation was introduced with an aim to reduce the hepatotoxicity of VPA [25]. As mentioned earlier, metabolic conversion of VPA into (E)-2,4-diene-VPA via CYP450 and mitochondrial beta-oxidation results in further toxic intermediates, which causes hepatotoxicity. The introduction of fluorine at the alpha carbon of VPA, forms α-fluoro-4-ene VPA upon metabolic conversion [91]. This intermediate is a non-toxic analogue of 4-ene VPA and does not undergo beta-oxidation, thus preventing the formation of downstream hepato-toxic metabolite, 2,4-diene-VPA [92]. This non-toxic effect was evaluated in rats administered with α-fluoro-4-ene VPA, wherein no signs of hepatic microvesicular steatosis were observed. The derivative also showed reduced teratogenic effect in NMRI mice compared to VPA [61].

Although α-fluoro VPA did show its suppressive effect against PTZ-induced seizures in CD-1 mice, its maximal effect was much delayed likely due to its slower brain uptake [24]. Its ED_50_ value was observed to be 275.74 mg/kg vs. that of VPA with an ED_50_ of 119.7 mg/kg [24].

### 3.4. Cyclic Analogues of VPA

#### 3.4.1. Cyclooctylideneacetic Acid (2-Cycloctylideneacetic Acid)

Cyclooctylideneacetic acid is a 168.23 g/mol cyclic acid analogue of VPA. In a study by Palaty J and Abbott F.S., mice with PTZ-induced seizure were subjected to 17 VPA-related cyclic and acyclic analogues. Of these 17 analogues, cyclooctylideneacetic acid was found to be the most potent anticonvulsant, even exceeding the potency of VPA with an ED_50_ dose (122.8 mg/kg) lower than VPA [55]. Moreover, the compound imparted a significantly reduced sedative effect on the treated mice at the observed ED_50_ value [55].

#### 3.4.2. Tetramethylcyclopropyl Analogues

These analogues of VPA were designed to inhibit the conversion of VPA to its hepatotoxic metabolites [93]. The lack of potential transformation of this analogue is due to presence of two tertiary carbons at beta position to the carboxyl moiety [64].

##### 2,2,3,3-Tetramethylcyclopropanecarboxylic Acid (TMCA)

TMCA is a 142 g/mol cyclopropyl analogue of VPA. It has weak anticonvulsant activity as compared to VPA, but has enhanced antiallodynic activity compared to parent drug, at doses that do not show any sedation effect [62]. This was shown in SNL mice model for tactile allodynia where the antiallodynic effect was observed at ED_50_ value of 181 mg/kg vs. ED_50_ value of 269 mg/kg of VPA 60 min post dosage [62].

In a fashion similar to the production of VPA amides with improved potency, TMCA amide derivatives were also studied as discussed in below sections.

##### 2,2,3,3-Tetramethylcyclopropanecarboxamide (TMCD)

TMCD is a 141 g/mol cyclopropyl analogue of VPD. Okada et al. observed the analogue to have better anticonvulsant activity and lesser teratogenicity in mice embryos than VPA [64]. Consistent finding was also reported in rats, where reduced fetal skeletal abnormalities were observed as compared to VPA [63]. Hence, the analogue was suggested as a possible candidate for VPA replacement. Similar to TMCA, TMCD was also found to be potent antiallodynic drug with ED_50_ of 85 mg/kg [62].

##### N-Methoxy-TMCD (MTMCD)

It is a 154 g/mol derivative of TMCD, where the hydrogen atom of NH_2_ in TMCD is substituted with a methoxy group. MTMCD is a broad-spectrum anticonvulsant [63,94]. In the same study by Okada et al., enhanced anticonvulsant activity for MTMCD in comparison to VPA was observed, accompanied with less teratogenicity shown in vivo [64]. This compound was found to have 18.5 times more potency than VPA in rat subcutaneous metrazol (scMet) test and 4.5 times in MES test. It also has a wider safety margin (8 times the protective index (PI = TD_50_/ED_50_) of VPA) [64]. Among other cyclopropyl analogues of VPA, MTMCD showed best antiallodynic activity with ED_50_ value of 41 mg/kg [62].

##### Alpha-Fluro-TMCD

As mentioned, fluorinated VPA analogues show reduced hepatotoxicity but were also accompanied by reduced anticonvulsant activity [24,25,92]; this limitation was overcome by fluorination of TMCD [65]. Alpha-fluoro TMCD showed 120 times more potent anticonvulsant activity in scMET rat model than VPA [65]. It also showed higher potency in other in vivo test (kindled rat model, 6 Hz test and pilocarpine induced seizure in rat) and was found to be non-teratogenic with better safety margin than VPA, due to which it was proposed to be a potent candidate of CNS therapeutics [65].

Other compounds in this category are cyclohexanecarboxylic acid and its methylated form. Cyclohexanecarboxylic acid, a 128.17 g/mol analogue, showed promise with reduced neurotoxicity, though it still maintains the low potency of VPA [90]. In contrast, the 142.2 g/mol methylated analogue, i.e., 1-methylcyclohexane carboxylic acid depicted higher potency, but had a fatally increased neurotoxic potential [90].

### 3.5. Urea Derivatives of VPA

In the studies targeted at understanding the structure–activity relationships between VPA and its adverse effects, a number of urea derivatives have also been synthesized. The three compounds showing promise as future anticonvulsants are derived from VPA amide derivatives, and have not only improved anticonvulsant activity but also broader spectrum [56].

#### 3.5.1. Valnoctylurea (VCU, 2-Ethyl-3-methylpentanoyl Urea)

VCU is a 186 g/mol urea derivative of VCA. VCU along with diisopropyl acetic acid (DIU) has shown tremendous anticonvulsant potential in scMET and MES seizure model, even better than amide derivatives of VPA [56]. The observed ED_50_ values for VCU are 14 mg/kg and 48 mg/kg in scMET seizures and 6Hz seizures, respectively, which was much better than those observed for VPA (646 mg/kg and 310 mg/kg respectively) [56]. In addition, VCU also has a lower teratogenic effect, leading to its acceptance as a safer derivative of VPA [56].

#### 3.5.2. Propyl Isopropylacetyl Urea (PIU)

PIU is a 186 g/mol urea derivative of PID with the characteristic C-2 chiral centre. The enantioselective anticonvulsant effect of (R) and (S)-PIU was consistently observed in both MES and scMET-induced seizures [56]. In MES model, an ED_50_ value of 16 mg/kg was determined for the racemic mixture of PIU enantiomers, whereas individual R-PIU and S-PIU enantiomers had ED_50_ 36 mg/kg and 18 mg/kg respectively [56]. Opposite results were observed in scMET model, where (R)-PIU had better efficacy with ED_50_ of 22 mg/kg that was significantly lower than S-PIU (ED_50_ = 37 mg/kg) and racemic-PIU (ED_50_ = 45 mg/kg) [56]. Results similar to scMET model was also observed for 6Hz model.

Teratogenic effect of (R)-PIU was observed at doses 5 to 6 times higher than the its ED_50_ [56]. This effect was greater than the S enantiomer. The significant difference in the toxicity of the two enantiomers can be ascertained as (R)-PIU has 54% embryotoxicity as compared to 10% of (S)-PIU [56]. The R isomer also leads in causing neural tube defects in the exposed embryos [56]. Yet the safety margin of both the enantiomers was better than VPA at their effective doses.

#### 3.5.3. Diisopropyl Acetyl Urea (DIU)

DIU is a 186 g/mol urea derivative of diisopropyl acetyl amide (DIA). DIU has shown itself to be a promising anticonvulsant against 6Hz seizures with ED50 of 49 mg/kg that is sufficiently lower than VPA (310 mg/kg) [56]. It also showed better results than VPA in scMET and MES model with ED_50_ value of 16 mg/kg and 33 mg/kg, respectively, compared to ED_50_ value of 646 mg/kg and 485 mg/kg of VPA. Like other urea derivatives of VPA amide, DIU has better safety margin than VPA [56].

### 3.6. Conjugation Products of VPA

#### Valrocemide (TV1901, VGD)

VGD is a 200.28 g/mol amide derivative conjugation product of VPA and glycinamide. The conjugate was targeted to maximize the brain penetration [95]. Drug-distribution study performed by Blotnik et al. documented 4 times higher brain-to-plasma ratio of VGD than VPA in rats, thus implying better brain distribution of the conjugate [96]. VGD turned out to be a VPA-like broad-spectrum anticonvulsant [57]. VGD has proven itself to show doubly potent anticonvulsant activity than VPA and can effectively counter MES, 6-Hz psychomotor seizures (even at 44 mA stimulation), PTZ-, picrotoxin-, and bicuculline-induced seizures in rat model. VGD lagged behind VPA only in managing seizures of hippocampal kindled rats. The compound has reached Phase II clinical trials as an AED for patients with refractory epilepsy [97].

VGD also lacks a carboxylic moiety, thus its teratogenic potential is essentially missing and, therefore, no teratogenic effect of the compound was observed in rats, rabbits or SWV mice [57].

### 3.7. Prodrugs (Sugar Esters of VPA)

Unlike the other compounds discussed above, the sugar esters do not aim to change the VPA-related adverse drug reactions (ADRs). Sugar esters as prodrugs of VPA were developed as slow-release formulations of VPA to counter its limited plasma half-life, though they were also found to exert direct action on the brain. Amongst these esters, dimethylenexylitol valproate (VDMX) turned up as the most potent compound with its effective dose as low as 100 times that of VPA in suppressing spontaneous epileptiform activities (SEA) in rat hippocampal slices [60]. Also, VDMX showed best protective index in PTZ test [60]. VDMX shows good activity in entorhinal cortex late recurrent discharges (LRD) model for pharmacoresistant epilepsy, even though VPA fails to exert any significant effect on suppressing epileptiform activity in LRDs [59]. It was speculated that since LRD primarily depend on glutamatergic mechanisms, VDMX might strongly affect glutamatergic synaptic transmissions to exert its actions.

## 4. Conclusions

Introduced in clinical practice about 60 years ago, valproic acid still represents one of the most efficient AED targeting broad spectrum of epilepsy types. Emerging evidence also demonstrates its use in other neurological disorders such as migraine, bipolar disorder and neuropathic pain. However, its use in pregnant women with epilepsy (WWE) has shown to be associated with teratogenic effects on the developing foetus leading to congenital abnormalities. In addition, hepatotoxicity is another adverse effect commonly experienced with VPA therapy.

Numerous attempts have been made by various research groups to modify VPA structure. These were focused on altering one of the factors deemed responsible for its teratogenic behaviour or on avoiding the formation of its hepatotoxic metabolites (4-ene-VPA and 2,4-diene-VPA) while simultaneously increasing or maintaining its anticonvulsant activity and potency. This review has meticulously accounted for all the structural derivatives and analogues of VPA, discussing their pharmacological and toxicological profiles tested in vivo. These included amide derivatives, acid analogues, fluorinated derivatives, cyclic analogues, urea derivatives, conjugation products and sugar esters. The modifications thus made, have proven themselves to be effective as anxiolytics, antiallodynics and better anticonvulsants than VPA. Furthermore, the majority of them have shown better potency and tolerability in vivo.

Among the developed amide derivatives, VCD and SPD were found to have potentially no teratogenicity, are broad spectrum AEDs just like VPA, and are under active investigations. VCD has shown itself to be non-teratogenic at therapeutic doses in animal models [98,99] and has an anticonvulsant profile comparable to SPD. Advantages of SPD over VCD are lower ED_50_ values in pilocarpine induced status epilepticus [66,100,101]. Even though the fluorinated derivatives of VPA have reduced hepatotoxicity and teratogenicity, they are not as effective anticonvulsants as VPA, thus stopping their development. A fluorinated hydroxamic acid derivative of; VPA 2-Fluoro-VPA-HA [61] and alpha-Fluoro-TMCD [65] proved to be good candidates as second generation VPA. Apart from this, effectiveness of VGD in controlling mouse 44mA 6Hz-induced seizures and its better brain distribution has led to its successful completion of a 13-week, phase II clinical trial in Europe with resistant patients with epilepsy [57,58,77].

This article provides necessary knowledgebase of existing modifications which may facilitate more rational attempts in making structural changes. These strategies will systematically enhance the therapeutic use of VPA-related compounds for better disease management.

## Figures and Tables

**Table 1:** Valproic acid analogues and derivatives, classification and pharmacological activity.

S. No	Candidate Compound	Parent Compound	Classification	Chemical Formula	Molecular Weight (g/mol)	Anticonvulsant Property	Clinical Trial Phase	Teratogenicity	Hepatotoxicity	Reference
1	Isovaleramide	Isovaleric acid	Aliphatic amide	C5H11NO	101.149	Reduced	Phase I, ^a^ Phase II	Reduced	N.D.	[49]
2	Valpromide	Valproic acid	Aliphatic amide	C8H17NO	143.23	Increased	NA	* No change in humans	* No change in humans	[50]
3	Valnoctamide	Valpromide	Aliphatic amide	C8H17NO	143.23	Increased	^b^ Phase III completed	Reduced	Reduced	[50]
4	sec-Butyl-propyl acetamide	Valpromide	Aliphatic amide	C9H19NO	157.25	Increased	Phase II	Reduced	Reduced	[51]
5	Propyl isopropyl acetamide	Valpromide	Aliphatic amide	C8H17NO	143.23	Increased	NA	Reduced	N.D.	[52]
6	2-ene-VPA	Valproic acid	Acid analogue	C8H14O2	142.2	Similar	^c^ Phase I	Reduced	Reduced	[53]
7	4-ene-VPA	Valproic acid	Acid analogue	C8H14O2	142.2	Similar	NA	Increased	Increased	[27,54]
8	Cyclooctylideneacetic acid	Valproic acid	Cyclic analogue	C10H16O2	168.23	Increased	NA	N.D.	N.D.	[55]
9	Cyclohexane carboxylic acid	Valproic acid	Cyclic analogue	C7H12O2	128.17	Similar	NA	Reduced	N.D.	[55]
10	1-methyl cyclohexane carboxylic acid	Cyclohexane carboxylic acid	Cyclic analogue	C8H14O2	142.2	Increased	NA	N.D.	N.D.	[55]
11	Valnoctyl urea	Valnoctic acid	Urea Derivative	C9H18N2O2	186.25	Increased	NA	Reduced	N.D.	[56]
12	Propyl isopropylacetyl urea	Diisopropyl acetamide	Urea Derivative	C9H18N2O2	186.25	Increased	NA	Reduced	N.D.	[56]
13	Diisopropyl acetyl urea	Valproic acid	Urea Derivative	C9H18N2O2	186.25	Increased	NA	N.D.	N.D.	[56]
14	Valrocemide	Valproic acid	Conjugation product	C10H20N2O2	200.282	Increased	^d^ Phase II	Absent	N.D.	[57,58]
15	Dimethylenexylitol valproate	Valproic acid	Sugar ester	C15H26O6	302.36	Increased	NA	N.D.	N.D.	[59,60]
16	α-Floro-VPA	Valproic acid	Acid analogue	C8H15O2F	162.20	Reduced	NA	Reduced	Reduced	[61]
17	TMCA	Valproic acid	Cyclic analogue	C8H14O2	142.20	Reduced	NA	N.D.	N.D.	[62]
18	TMCD	TMCA	Cyclic analogue	C8H15NO	141	Increased	NA	Reduced	N.D.	[62,63]
19	MTMCD	TMCA	Cyclic analogue	C9H17NO	154	Increased	NA	Reduced	N.D.	[63,64]
20	α-Floro-TMCD	TMCA	Cyclic analogue	C8H14FNO	159.20	Increased	NA	Reduced	N.D.	[24,65]

Note—The change in anticonvulsant activity, teratogenicity and hepatotoxicity is mentioned with respect to valproic acid. * Since valpromide transforms into valproic acid in humans, there is no change in teratogenicity for valpromide; NA—not applicable, these compounds did not enter the clinical trials; N.D.—not determined; ^a^: for acute migraine headache; ^b^: for mania, schizoaffective disorder, manic type; ^c^: drug tolerance study in health volunteers; ^d^: for therapy-resistant patients with epilepsy.

## Abbreviations

VPA: valproic acid: CNS, central nervous system; AED, antiepileptic drug; GABA, gamma-aminobutyric acid; OAT, organic anion transporters; CYP450, cytochrome P450; GABA-T, GABA transaminase; SSADH, succinate semialdehyde dehydrogenase; GluR1/2, glutamate receptor subunits 1 and 2; SCN3A, sodium voltage-gated channel alpha subunit 3; HDAC, histone deacetylase; GABAR: GABA receptors; BDNF: brain derived neurotrophic factor; CCND2: cyclin D2; PGE2: prostaglandin E2; GSK3B: glycogen synthase kinase 3 beta; PKC: protein kinase C; ERK/BCL-2: extracellular regulated kinase—B-cell lymphoma 2 pathway; Bad: BCL2 associated agonist of cell death; CDK5: cyclin-dependent kinase 5; NEP: neprilysin; TGF-B: transforming growth factor beta; VEGF: vascular endothelial growth factor, IL-1B: interleukin 1 beta; CCR1: C-C chemokine receptor type 1; p-JNK: phosphorylated c-Jun N-terminal kinase; H3K9ac: histone 3 lysine 9 acetylation; Ngb: neuroglobin; Nrf2/HO-1: nuclear factor erythroid 2–related factor 2—heme oxygenase-1 pathway; H3ac: histone 3 acetylation; H4ac: histone 4 acetylation; p21: cyclin-dependent kinase inhibitor; MYC: master regulator of cell cycle entry and proliferative metabolism; CCND1: cyclin D1; NOTCH1: Notch homolog 1, translocation-associated; p63: tumor protein p63; IL-6: interleukin 6; ANF: atrial natriuretic peptide; MES, maximal electroshock stimulation; PTZ, pentylenetetrazole; VPD, valpromide; SOX9, SRY-Box Transcription Factor 9; RUNX2, Runt-related transcription factor 2; VCD, valnoctamide, VCA, valnoctic acid; SE, status epilepticus; SNL, spinal nerve ligation; Pc-G, Polycomb group; NMRI, Naval Medical Research Institute; SPD, sec-Butyl-propylacetamide; ED_50_, effective dose; NTD, neural tube defect; PID, propylisopropyl acetamide; GTCS, generalized tonic–clonic seizures; TMCA, 2,2,3,3-tetramethylcyclopropanecarboxylic acid; TMCD, 2,2,3,3-tetramethylcyclopropanecarboxamide; MTMCD, N-methoxy-TMCD; scMET, subcutaneous Metrazol; VCU, valnoctylurea; DIU, diisopropyl acetyl urea; DIA, diisopropyl acetic acid. PIU, propyl isopropylacetyl urea; VGD, valrocemide; ADR, adverse drug reaction; SEA, spontaneous epileptiform activities; VDMX, dimethylenexylitol valproate; LRD, late recurrent discharges; WWE, women with epilepsy.
